# C-954-06. Comparing Outcomes Among Frail and Non-Frail Patients Following Non-Operative Management in Older Adult Burn Patients

**DOI:** 10.1093/jbcr/irag033.138

**Published:** 2026-04-06

**Authors:** Andrew Bieterman, Colette Galet, David M Hill, Lauren T Moffatt, Jeffrey W Shupp, Kathleen S Romanowski, Shawn Tejiram

**Affiliations:** MedStar Washington Hospital Center, Washington, DC; University of Iowa, Iowa City, IA; Firefighters Regional One Burn Center, Memphis, TN; The Burn Center at MedStar Washington Hospital Center, Washington, DC; MedStar Washington Hospital Center, Washington, DC; UC Davis Burn Center & Shriners Hospital for Children, Sacramento, CA; MedStar Washington Hospital Center, Washington, DC

## Abstract

**Introduction:**

Frailty is increasingly recognized as a critical determinant of outcomes in older adult patients and is used to guide individualized care. Non-operative management (NOM) of older adult burn injured patients is often considered in the context of burn size, depth, and comorbidities. However, there is a paucity of literature examining outcomes using NOM, especially in the context of frailty. Herein, we compared outcomes of frail and non-frail older adult burn injured patients undergoing NOM.

**Methods:**

This is a retrospective multicenter cohort study. Burn injured patients 60 years and older admitted to 12 burn centers from 2017 to 2019 were included. Demographics, injury characteristics, and clinical metrics were obtained. Frailty was scored using the Canadian Study of Health and Aging Clinical Frailty Scale (CSHA-CFS). Patients were grouped as fit (Score < 4), pre-frail (Score = 4), and frail (Score > 4). Length of stay (LOS), long-term mortality, and discharge disposition were evaluated. Univariate and multivariate analyses were performed to compare groups and identify predictors of poor outcomes. *p*<.05 was considered significant.

**Results:**

Of 1528 older adult burn patients, 711 patients underwent NOM and had frailty scores. Of these, 255 patients were deemed fit, 160 were prefrail, and 296 were frail. There were no significant differences in sex, race, and ethnicity between the groups. Frail patients were significantly older than their prefrail and fit counterparts (69 [64-77] vs. 68 [62-76] vs. 66 [62-73], *p*<.001). Most patients suffered flame and flash burns (*p*=.008). No significant differences in median TBSA and inhalation injury were observed. The modified Baux score was highest among frail and prefrail patients (74.5 [67-85.9] vs. 75 [68-85.7] vs. 71.5 [65.6-81], *p*=.012). Frail patients had the highest LOS and LOS/%TBSA (*p*<.001). Long-term mortality rate was significantly higher in the frail group (27% vs. 3.8% vs. 2.7%, *p*<.001). Fit patients were more likely to be discharged home (86.9%), whereas frail patients were discharged to a skilled nursing facility (17.6%), inpatient rehabilitation (4.2%), and hospice care (5.7%) (*p*<.001). On multivariate analysis, modified Baux score and frailty status were associated with in-hospital mortality (OR = 1.13 [1.09-1.17] and 4.92 [1.09-22.11]) and hospice disposition (OR = 1.08 [1.02-1.14] and 19.68 [5.03-77]).

**Conclusions:**

Burn injured patients who underwent NOM and were identified as either pre-frail or frail experienced higher mortality, LOS, and discharge disposition requiring higher level of care. Further study of this novel multicenter study on frailty and NOM will be necessary to better elucidate observed outcomes.

**Applicability of Research to Practice:**

Understanding the effect of frailty on burn NOM strategies provides an important framework on understanding outcome variability and guiding individualized care.

**Funding for the study:**

N/A.

